# Fluctuation of functional somatic disorders in a population-based cohort. The DanFunD study

**DOI:** 10.1371/journal.pone.0312031

**Published:** 2024-10-16

**Authors:** Signe U. Schovsbo, Line L. Kårhus, Anne A. Bjerregaard, Marie W. Petersen, Lisbeth Frostholm, Per Fink, Tina B. W. Carstensen, Lene F. Eplov, Michael E. Benros, Susanne Brix, Anja L. Madsen, Allan Linneberg, Thomas M. Dantoft, Torben Jørgensen

**Affiliations:** 1 Center for Clinical Research and Prevention, Copenhagen University Hospital – Bispebjerg and Frederiksberg, Copenhagen, Denmark; 2 The Research Clinic for Functional Disorders and Psychosomatics, Aarhus University Hospital, Aarhus, Denmark; 3 Department of Clinical Medicine, University of Aarhus, Aarhus, Denmark; 4 Copenhagen Research Center for Mental Health – CORE, Mental Health Centre Copenhagen, Copenhagen, Denmark; 5 Department of Clinical Medicine, Faculty of Health and Medical Science, University of Copenhagen, Copenhagen C, Denmark; 6 Department of Biotechnology and Biomedicine, Technical University of Denmark, Kongens Lyngby, Denmark; 7 Department of Public Health, Faculty of Health and Medical Science, University of Copenhagen, Copenhagen C, Denmark; Northwestern University Feinberg School of Medicine, UNITED STATES OF AMERICA

## Abstract

**Background:**

Evidence of incidence of functional somatic disorders (FSD) is hampered by unclear delimitations of the conditions and little is known about the possible interchangeability between syndromes. Further, knowledge on remission and persistence of FSD in the general population is limited. We aimed to assess the natural course of various FSD over 5 years in the general population.

**Methods:**

A follow-up study (Danish Study of Functional Disorders—DanFunD) was conducted in a random sample of the general population comprising 5,738 participants aged 18–76 years at baseline. Both at baseline and five-year follow-up, participants filled in validated questionnaires on symptoms to delimitate two approaches of FSD, the bodily distress syndrome (BDS) and four functional somatic syndromes (FSS): irritable bowel (IB), chronic fatigue (CF), chronic widespread pain (CWP), and multiple chemical sensitivity (MCS).

**Results:**

Both BDS and FSS showed a five-year incidence around 11%. Incidence of the individual FSS varied from 0.8% (MCS) to 5.7% (CF). BDS and FSS showed a remission proportion close to 50%. We found a high degree of interchangeability between each FSS varying from 15.0% to 23.4%.

**Conclusion:**

We identified a marked fluctuation pattern of FSD during a five-year period, with a high degree of interchangeability between each FSS. The study stresses the importance of large population-based cohorts with transparent delimitation of FSD in future research to understand these complex conditions.

## Introduction

Functional somatic disorder (FSD) is a diagnosis including various conditions such as functional somatic syndromes (FSS), e.g., irritable bowel syndrome (IBS), fibromyalgia or chronic widespread pain (FM/CWP), chronic fatigue syndrome (CFS), multiple chemical sensitivity (MCS), and the unifying diagnostic construct bodily distress syndrome (BDS) [1, 2]. Common for the conditions are persistent and troublesome physical symptoms presenting in characteristic symptom patterns. The symptoms are believed to be products of both biological and psychosocial factors and are often accompanied by impairment or disability [3].

Literature reviews have shown a great variation in methods of measurements and in incidence of FSS with large interquartile ranges of IBS (38.5 cases per 10,000 person years (interquartile range = 20–45.3)) [4], FM (4.3 cases per 1,000 person years (range 0.33–18.8)) [5], and CWP (12.5 cases per 1,000 person years (range 7.2–81.6)) [5]. Incidence of CFS was in one study 0.2% at 2.4 years follow-up [6] and in two other studies it was estimated to be from 25.8 to 370 cases per 100,000 person years [7, 8]. In a cohort study in primary care, the incidence of BDS was 6.9% over two years, and 56.8% of baseline cases persisted at follow-up [9].

Delimitation of the various FSD in the literature varies considerably from diagnosis based on self-report over a doctor’s diagnosis and ICD-codes to a variety of symptoms used for delimitation of the syndrome in question [4, 5, 10]. Prior studies are often not specifically designed for FSD and rarely address more than one FSD in the same study. Consequently, the incidence could cover both true incidence and a change from one type of FSD to another, as a large overlap is seen between the syndromes [11]. These methodological differences might explain the great variation in the reported incidence. A more global aspect of the natural history of FSD should therefore include both incidence, persistence, and remission of symptoms, which has been done in only a few studies [9, 12–14]. These indicate that FSD represent fluctuational syndromes. However, large cohort studies from the general population taking several FSD into account simultaneously are warranted.

Utilizing the large population-based cohort established in the Danish Study of Functional Disorders (DanFunD), we aimed to assess the natural course of FSD over 5 years using internationally accepted delimitations for various FSD.

## Materials and methods

### Study population

The DanFunD baseline cohort was established in 2011–2015 and consists of a part 1 (n = 2,308) and a part 2 (n = 7,493) [15–17]. DanFunD part 1 was conducted as a 5-year follow-up investigation of the Health2006-cohort carried out in 2011–2012, whereas DanFunD part 2 was carried out in 2012–2015. DanFunD part 2 included a new random sample selected through the National Civil Registration system among people living in the same municipalities in the western part of greater Copenhagen, Denmark as persons recruited to Health2006. Thus, all participants were invited between 11/02/2011 to 06/08/2015 and examined between 11/10/2011 and 06/30/2015. A total of 9,656 unique participants constitutes the DanFunD baseline cohort. The participants were all born in Denmark and were between 18 and 76 years of age at baseline. Participants filled in a questionnaire in paper and went through a health examination at the center conducted by trained nurses.

In the period of 13/03/2018 to 21/10/2020 all eligible participants from baseline (n = 9,323) were invited for a follow-up study, and a total of 5,738 (61.5%) participated between 22/03/2018 and 04/01/2021. All participants were followed with self-administered electronic questionnaires only, whereas participants of DanFunD part 2 also completed a general health examination conducted by trained nurses. Median follow-up period was 65 months (range of 47–108 months).

#### Ethics approval

The study was conducted in accordance with the Helsinki II declaration and approved by the Ethical Committee of Copenhagen County (Ethics Committee: H-3-2011-081; H-3-2012-0015; H-17021141) and the Danish Data Protection Agency. Written informed consent was obtained from each participant before participation.

### Outcomes: Functional somatic disorders

Using validated questionnaires, delimitation of FSD were based on two different approaches. One approach was the BDS-concept. BDS is defined by the 25-item BDS checklist [18] and comprises four symptom clusters: cardiopulmonary (CP), gastrointestinal (GI), musculoskeletal (MS), and general symptoms (GS). Based on the number of involved symptom clusters, BDS was assigned as single-organ BDS (at least four symptoms within one or two of the symptom clusters) or multi-organ BDS (at least four symptoms from at least three of the symptom clusters).

In the other approach four common delimitations of FSS were used. We avoid using the word syndrome due to current disagreements on terminology and criteria. Irritable bowel (IB) was defined using the definition by Kay and Jørgensen [19]; chronic fatigue (CF) was defined using the definition by Chalder et al. [20]; chronic widespread pain (CWP) was defined using the American College of Rheumatology Criteria [21] and the definition by White et al [22]; and multiple chemical sensitivity (MCS) was defined using an adaptation of the 1999 consensus definition [23], with modifications by Lacour et al. [24, 25]. Participants with IB, CF, CWP, or MCS without any of the other three delimitations were assessed in individual analyses [11].

The different delimitations in DanFunD such as BDS [2] and FSS [11] have been found to overlap and capture the same groups of participants, also after exclusion of self-reported chronic physical disease [26]. The prevalence was found to be 16.3% for FSS and 16.1% for BDS at DanFunD baseline [27].

Incidence, remission, and persistence were assessed for both approaches; BDS (total, single- and multi-organ) and the FSS. The FSS delimitations were assessed both with and without comorbid FSS delimitations. Incidence was defined as cases without a specific delimitation at baseline, but presence at follow up, whereas remission was defined as cases with a specific delimitation at baseline, but no type of delimitation at follow-up. Persistence was defined as cases having the same delimitation at baseline and follow-up. Interchangeability was defined as having one delimitation at baseline and another at follow-up. See S1 and S2 Figs. for overview of the definitions.

### Statistical analysis

All analyses were conducted using SAS Guide Enterprise version 7.1. Baseline characteristics were presented for both participants and non-participants at follow-up as percentage or mean and standard deviation. The differences in baseline characteristics between participants and non-participants at follow-up was analyzed using t-test and chi square analyses calculating p-values at a 5% significance level.

The five-year incidence, remission, and persistence were expressed as proportions (in percent with 95% CI) of the participants at risk over 5 years. Sex and age differences by age groups (18–45 years, 46–60 years, 60+ years) were analyzed using chi square and Fisher’s exact test and expressed by p-values. Significance level was 5%.

All analyses were conducted as complete case-analyses; thus, participants should have complete information on both the specific FSD type at baseline and the specific FSD type at follow up to be included in the individual analyses.

## Results

A total of 5,738 persons participated in the follow-up study; mean age at baseline was 54 years and 53% were women. Non-participation in the follow-up investigation was associated with younger age, female sex, and FSD at baseline (Table 1).

**Table 1:** Characterization of the 9,323 DanFunD participants attending and not attending the follow up-study.

	Participants in follow up	Non-participants follow up	p-value
	% (n/N)	% (n/N)	
Participants (N = eligible for follow up)	61.5 (5738/9323)	38.5 (3585/9323)	
**Age at baseline**			
Mean age in years at baseline (std)	54.1 (11.8)	49.7 (14.7)	**< .001**
Age groups at baseline			
18–45 years	21.8 (1249/5738)	36.6 (1311/3585)	
46–60 years	43.2 (2476/5738)	35.3 (1265/3585)	
+60 years	35.1 (2013/5738)	28.2 (1009/3585)	
**Sex at baseline**			
Men	46.7 (2678/5738)	44.3 (1589/3585)	**0.027**
Women	53.3 (3060/5738)	55.7 (1996/3585)	
**Bodily Distress Syndrome at baseline**			
No	86.1 (4924/5716)	80.3 (2839/3536)	**<0.001**
Yes	13.9 (792/5716)	19.7 (697/3536)	
**IB, CF, CWP or MCS at baseline**			
No	86.8 (4831/5565)	82.7 (2813/3403)	**< .001**
Yes	13.2 (734/5565)	17.3 (590/3403)	

Abbreviations: IB = Irritable bowel, CF = Chronic fatigue, CWP = Chronic widespread pain, MCS = Multiple chemical sensitivity, std = standard deviation. N varies as the analyses were conducted as complete cases analyses.

### Incidence

The five-year incidence for BDS was 11.2% (Table 2). This was primarily due to single-organ BDS, whereas multi-organ BDS had a lower incidence of 0.6%. Female sex showed the highest incidence, whereas the trend towards higher incidence in younger age was not significant.

**Table 2:** Incidence of bodily distress syndrome and functional somatic syndromes in the DanFunD cohort (N = 5,738).

	Participants included for analysis N	Total % (95% CI)	Sex			Age (years)			
			Men % (95% CI)	Women % (95% CI)	p-value	18–45% (95% CI)	46–60% (95% CI)	+60% (95% CI)	p-value
**Bodily distress syndrome**									
No BDS → total BDS	4881	11.2 (10.3–12.1)	8.7 (7.5–9.8)	13.7 (12.3–15.0)	**< .001**	13.3 (11.3–15.3)	10.7 (9.4–12.0)	10.5 (9.1–12.0)	0.052
No BDS → single BDS	4881	10.6 (9.8–11.5)	8.2 (7.1–9.3)	13.0 (11.7–14.3)	**< .001**	12.5 (10.6–14.5)	10.1 (8.9–11.4)	10.0 (8.6–11.4)	0.066
No BDS → multi BDS	4881	0.6 (0.4–0.8)	0.5 (0.2–0.8)	0.8 (0.4–1.1)	0.240	0.7 (0.2–1.2)	0.6 (0.3–0.9)	0.5 (0.2–0.9)	0.799
**Functional somatic syndromes**									
No CWP → CWP	5409	4.8 (4.2–5.4)	3.5 (2.8–4.2)	6.0 (5.1–6.9)	**< .001**	2.7 (1.8–3.6)	5.3 (4.4–6.2)	5.5 (4.5–6.6)	**0.001**
No CF → CF	5228	5.7 (5.1–6.4)	3.8 (3.0–4.5)	7.6 (6.6–8.5)	**< .001**	10.2 (8.4–12.0)	4.7 (3.8–5.6)	4.4 (3.4–5.3)	**< .001**
No IB → IB	5400	2.7 (2.3–3.2)	1.3 (0.9–1.7)	4.0 (3.3–4.7)	**< .001**	4.0 (2.9–5.1)	3.1 (2.4–3.8)	1.4 (0.9–2.0)	**< .001**
No MCS → MCS	5519	0.8 (0.5–1.0)	0.5 (0.2–0.8)	1.0 (0.6–1.3)	**0.042**	0.6 (0.2–1.0)	0.8 (0.4–1.1)	0.9 (0.5–1.3)	0.610
**Functional somatic syndromes without comorbid FSS**									
No FSS → any FSS *	4780	10.5 (9.6–11.4)	7.6 (6.5–8.7)	13.3 (11.9–14.6)	**< .001**	13.4 (11.3–15.4)	9.9 (8.6–11.2)	9.4 (8.0–10.8)	**0.003**
No FSS → only CWP**	4780	3.0 (2.5–3.5)	2.7 (2.1–3.4)	3.3 (2.5–4.0)	0.289	1.1 (0.4–1.7)	3.2 (2.4–4.0)	3.9 (3.0–4.9)	**< .001**
No FSS → only CF**	4780	4.2 (3.6–4.8)	3.2 (2.5–3.9)	5.1 (4.3–6.0)	**0.001**	8.0 (6.4–9.7)	3.1 (2.3–3.8)	3.2 (2.4–4.1)	**< .001**
No FSS → only IB**	4780	1.6 (1.3–2.0)	0.8 (0.4–1.1)	2.4 (1.8–3.0)	**< .001**	2.4 (1.5–3.4)	1.8 (1.3–2.4)	0.8 (0.4–1.3)	**0.003**
No FSS → only MCS**	4780	0.4 (0.3–0.6)	0.4 (0.1–0.6)	0.5 (0.2–0.8)	0.565	0.4 (0.0–0.8)	0.4 (0.1–0.7)	0.5 (0.2–0.9)	0.815

Abbreviations: BDS = Bodily distress syndrome, Single BDS = single-organ bodily distress syndrome, Multi BDS = multi-organ bodily distress syndrome, FSS = umbrella term for CWP, CF, IB and MCS, CWP = chronic widespread pain, CF = chronic fatigue, IB = irritable bowel, MCS = multiple chemical sensitivity. CI = confidence interval. N = total number of participants at risk. N varies as the analyses were conducted as complete case analyses, thus participants had data on specific syndrome at both baseline and follow-up. Differences in sex and age were analyzed by chi-square and Fisher’s exact test. Numbers marked with bold are differences with a p-value <0.05

*: Any FSS (CWP, CF, IB, MCS),

**: Neither of the three other FSS.

The incidence of any of the four FSS was 10.5% with a preponderance of women and younger persons (Table 2). The incidence of MCS, IB, CWP, and CF, allowing for comorbid FSS, were 0.8%, 2.7%, 4.8%, and 5.7%, respectively with a female preponderance. The same order from lowest to highest, though with lower values, was observed when assessing MCS, IB, CWP, and CF without comorbid FSS. A female preponderance was found only for CF and IB. Incidence of CF and IB was highest in the younger age-groups, whereas for CWP it was highest in the older age-groups both with and without comorbid FSS.

### Remission

Remission of total BDS was observed in 44% of the cases and among persons with multi-organ BDS, 10.8% showed remission (Table 3). Remission was observed in 46% of FSS cases and was associated with male predominance for both BDS and FSS. Remission of CWP and CF was higher for males than females, whereas no sex difference was seen for IB and MCS. Age was not associated with remission.

**Table 3:** Remission of bodily distress syndrome and functional somatic syndromes in the DanFunD cohort (N = 5,738).

	Participants included for analysis N	Total % (95% CI)	Sex			Age (years) at baseline			
			Men % (95% CI)	Women % (95% CI)	p-value	18–45% (95% CI)	46–60% (95% CI)	+60% (95% CI)	p-value
**Bodily distress syndrome**									
Total BDS → no BDS	782	44.0 (40.5–47.5)	50.8 (44.6–57.0)	40.8 (35.6–44.9)	**0.008**	42.3 (34.1–50.6)	45.7 (40.5–50.8)	42.7 (36.9–48.4)	0.679
Single BDS → no BDS	745	45.6 (42.1–49.2)	52.9 (46.6–59.2)	42.2 (37.8–46.5)	**0.006**	45.3 (36.7–53.9)	47.8 (42.4–53.1)	43.2 (37.4–49.0)	0.525
Multi BDS → no BDS	37	10.8 (0.8–20.8)	0.0	14.8 (1.4–28.2)	0.557	0.0	13.6 (0.0–28.0)	16.7 (0.0–46.5)	0.601
**Functional somatic syndromes**									
FSS → no FSS	718	46.2 (42.6–50.0)	54.3 (47.8–61.1)	42.9 (38.6–47.2)	**0.005**	43.7 (36.3–51.1)	46.2 (40.7–51.6)	48.4 (41.8–55.0)	0.647
CWP → no FSS	223	33.2 (27.0–39.4)	46.8 (32.5–61.1)	29.6 (22.8–36.3)	**0.026**	27.3 (8.7–45.9)	35.1 (26.3–43.9)	32.2 (22.4–42.0)	0.751
CF → no FSS	400	42.5 (37.7–47.3)	52.2 (43.0–61.4)	38.7 (33.0–44.3)	**0.014**	45.6 (36.7–54.3)	37.8 (30.9–44.8)	47.8 (37.5–58.1)	0.206
IB → no FSS	181	40.3 (33.2–47.5)	50.0 (36.1–63.9)	36.6 (28.9–44.9)	0.101	28.9 (16.5–41.2)	45.0 (34.1–55.9)	44.9 (31.0–58.8)	0.135
MCS → no FSS	94	50.0 (39.9–60.0)	51.4 (34.9–68.0)	49.2 (36.4–61.9)	0.831	33.3 (11.6–55.1)	55.6 (39.3–71.8)	52.5 (37.0–68.0)	0.280

Abbreviations: BDS = Bodily distress syndrome, Single BDS = single-organ bodily distress syndrome, Multi BDS = multi-organ bodily distress syndrome, FSS = umbrella term for CWP, CF, IB and MCS, CWP = chronic widespread pain, CF = chronic fatigue, IB = irritable bowel, MCS = multiple chemical sensitivity. CI = confidence interval. N = total number of participants at risk. N varies as the analyses were conducted as complete case analyses, thus participants had data on specific syndrome at both baseline and follow-up. Differences in sex and age were analyzed by chi-square and Fisher’s exact test. Numbers marked with bold are differences with a p-value <0.05.

### Persistence

Persistence was observed in more than half of the BDS cases with a female predominance (Table 4). 5.8% of single-organ BDS cases at baseline changed to multi-organ BDS at follow-up, and more than half of persons with multi-organ BDS changed to single-organ BDS. No difference among the age-groups was observed.

**Table 4:** Persistence of bodily distress syndrome and functional somatic syndromes in the DanFunD cohort (N = 5,738).

	Participants included for analysis N	Total % (95% CI)	Sex			Age (years) at baseline			
			Men % (95% CI)	Women % (95% CI)	p-value	18–45% (95% CI)	46–60% (95% CI)	+60% (95% CI)	p-value
**Bodily distress syndrome**									
Total BDS → total BDS	782	56.0 (52.5–59.5)	49.2 (43.0–55.4)	59.3 (55.1–63.4)	**0.008**	57.7 (49.4–65.9)	54.3 (49.2–59.5)	57.3 (51.6–63.1)	0.679
Single BDS → single BDS	745	48.6 (45.0–52.2)	40.1 (33.9–46.3)	52.7 (48.3–57.1)	**0.001**	48.4 (39.8–57.1)	44.2 (38.9–49.5)	53.9 (48.1–59.8)	0.056
Single BDS → multi BDS	745	5.8 (4.1–7.5)	7.0 (3.8–10.2)	5.2 (3.2–7.1)	0.309	6.3 (2.1–10.4)	8.0 (5.1–10.9)	2.9 (0.9–4.8)	**0.023**
Multi BDS → multi BDS	37	29.7 (15.0–44.5)	40.0 (9.6–70.4)	25.9 (9.4–42.5)	0.442	44.4 (12.0–76.9)	27.3 (8.7–45.9)	16.7 (0.0–46.5)	0.601
Multi BDS → single BDS	37	59.5 (43.6–75.3)	60.0 (29.6–90.4)	59.3 (40.7–77.8)	1.000	55.6 (23.1–88.0)	59.1 (38.6–79.6)	66.7 (29.0–100)	1.000
**Functional somatic syndromes**									
FSS → FSS*	718	53.8 (50.1–57.4)	45.7 (39.0–52.5)	57.1 (52.8–61.4)	**0.005**	56.3 (49.0–63.7)	53.9 (48.4–59.3)	51.6 (45.0–58.2)	0.647
CWP → CWP	224	50.5 (43.9–57.0)	43.8 (29.7–57.8)	52.3 (44.9–59.7)	0.295	39.1 (19.2–59.1)	50.0 (40.8–59.2)	54.0 (43.6–64.5)	0.442
CF → CF	401	42.6 (37.8–47.5)	36.0 (27.2–44.8)	45.3 (39.5–51.1)	0.088	46.4 (37.7–55.1)	43.2 (36.1–50.4)	36.3 (26.4–46.1)	0.323
IB → IB	183	41.0 (33.9–48.1)	41.2 (27.7–54.7)	40.9 (32.5–49.3)	0.974	45.3 (31.9–58.7)	38.8 (28.1–49.4)	40.0 (26.4–53.6)	0.745
MCS → MCS	94	26.6 (17.7–35.5)	14.3 (2.7–25.9)	33.9 (21.8–46.0)	**0.038**	33.3 (11.6–55.1)	25.0 (10.9–39.1)	25.0 (11.6–38.4)	0.772
**Interchangeable functional somatic syndromes**									
CWP → either CF, IB, or MCS	223	16.1 (11.3–21.0)	8.5 (0.5–16.5)	18.2 (12.5–23.9)	0.123	31.8 (12.4–51.3)	14.9 (8.4–21.5)	13.8 (6.6–21.0)	0.107
CF → either CWP, IB, or MCS	401	15.0 (11.5–18.5)	12.3 (6.2–18.3)	16.0 (11.8–20.3)	0.343	8.0 (3.2–12.8)	18.9 (13.3–24.6)	16.5 (8.9–24.1)	**0.027**
IB → either CWP, CF, or MCS	182	18.7 (13.0–24.3)	9.8 (1.6–18.0)	22.0 (15.0–29.3)	0.055	25.0 (13.2–36.8)	16.3 (8.2–24.3)	16.0 (5.8–26.2)	0.384
MCS → either CWP, CF, or IB	94	23.4 (14.9–32.0)	34.3 (18.6–50.0)	17.0 (7.4–26.5)	0.055	33.3 (11.6–55.1)	19.4 (6.5–32.4)	22.5 (9.6–35.4)	0.516

Abbreviations: CWP = chronic widespread pain, CF = chronic fatigue, IB = irritable bowel, MCS = multiple chemical sensitivity, FSS = umbrella term for CWP, CF, IB and MCS, CI = confidence interval. N = total number of participants at risk. N varies as the analyses were conducted as complete case analyses, thus participants had data on specific syndrome at both baseline and follow-up. Differences in sex and age were analyzed by chi-square and Fisher’s exact test. Numbers marked with bold are differences with a p-value <0.05.

* Any FSS (CWP, CF, IB, MCS).

More than half of the FSS cases persisted with a female predominance (Table 4). Persistence of MCS was higher in females than males, whereas the other delimitations did not show sex difference. There were no age differences. A substantial interchangeability was seen between the four delimitations, highest for MCS, where nearly a quarter changed to one of the other three delimitations. There was no sex-difference, but a change from CF to one of the other three delimitations was more frequent in the older age-groups.

## Discussion

This study had two important findings. First, we showed a fluctuating pattern of the various FSD with a five-year incidence for both BDS and FSS of about 11% with a women preponderance. We found nearly 50% remissions with a male preponderance. Second, we showed that the various delimitations of FSS interchange between each other during a five-year period demonstrating the risk of misclassification of incidence, remission, and persistence when only looking at one delimitation at the time.

Comparison with existing literature is hampered by the wide range of different criteria used for delimitation of the various FSD. A clinical cohort with 2 years follow-up used the same diagnostic criteria for BDS and showed a lower incidence (6.9%), but similar remission (43.2%) [9]. Moreover, multi-organ BDS was more persistent than single-organ BDS [9], which was comparable with the present study. To our knowledge, no other studies have investigated the incidence and prognosis of BDS in population-based cohorts.

Regarding the incidence of IB there are several population-based cohorts [6, 28–31], however, it is difficult to compare their findings with the present study, as data mostly stems from physician-diagnosed IB registered in administrative or clinical databases [28–30] with considerable variation in diagnostic criteria, or from self-perceived diagnosis [6]. None of the studies considered the great overlap between the various delimitations. A comprehensive review [4] comprising 38 studies in general population samples showed a calculated incidence rate of 38.5 per 10,000 person years (IQR 20–45.3). In the present study, the five-year incidence of 2.7% and 1.6% for persons with or without co-morbid FSS over a median of 5.4 years, respectively, correspond to 50.4 and 29.8 cases per 10,000 person years. The Lifelines study [6] showed an incidence rate of 48.9 per 10,000 person-years allowing for co-morbid FSS. This is comparable with the incidence of IB (including co-morbid FSS) in the present study, but higher than the incidence of IB without co-morbid FSS in the present study. This difference probably stems from the fact that the Lifelines study did not investigate IB without co-morbid FSS. Most studies agree on a women preponderance in incidence, whereas the association to age varies. Both young and old age have been associated with IB depending on the geographical population with older age being a risk factor in studies from Taiwan, and younger age and female sex being a risk factors in studies from western countries [4]. An earlier population-based study from Denmark using the same diagnostic criteria as in the present study found a higher incidence and remission of IB after five years compared to the present study [12]. The study had a narrower age-interval and was performed in the 1980’ies. Another Danish population-based study from 2017 [31] also found a higher incidence and persistency. However, the study population was younger and recruited by an internet-based research institute hereby differing from the DanFunD population limiting comparison.

As regard incidence of CWP/FM a comprehensive review [5] comprising 37 prospective population-based cohort studies has shown a calculated incidence rate of 4.3 per 1,000 person-years with a variation between 0.33–18.8. Most studies were cohort studies using different criteria for delimitation of CWP/FM, which can be the reason for the large variation [14, 32, 33]. The incidence of 4.8% and 3.0% over a median of 5.4 years in the present study, correspond to 9 and 6 cases per 1,000 person years with and without co-morbid FSS, respectively. The incidence in the Lifelines study was markedly lower with an incidence rate of 20.6 per 10,000 person-years [6], which could be due to different criteria of CWP/FM. Female sex and older/middle age were risk factors, which is comparable with the present study. In two studies, remission of CWP was reported to be 43.1% at three-year follow-up [14] and 65.9% at seven-year follow-up [13], also in line with our findings.

For the incidence of CF, two register-based studies [7, 8] estimated an incidence rate between 25.8 and 370 per 100,000 person-years, and a population-based study of self-perceived CF [6] showed a incidence-rate of 8.8 per 10,000 person-years. This is considerably lower than the present study, where it can be calculated that incidence rates are 106 and 77 cases per 10,000 person years for person with or without co-morbid FSS, respectively. These differences indicate different delimitations and clinically approaches to the condition, as the two studies were either register-based [7] or based on participants self-perceived diagnosis [6] limiting comparison. In the register-based study [7], the incidence rate varies strongly with age with a peak in the age-group 10–19 years and in 30–39 years. This is in accordance with the present study, where incidence of CF was more frequent in the youngest age-group (18–45 years). Lastly, no studies on incidence of MCS were found.

Limited published data are available on the fluctuating nature of the different FSS. From the Lifelines cohort study, IB was mentioned as a predictor for FM and visa-versa [6, 10], whereas CF was not predicted by IB or FM and did not itself predict any of these two [6]. In a register-based study from Taiwan [34], it was shown that persons with FM had a higher risk of developing IB than persons without. Budtz-Lilly et. al showed in a follow-up study in primary care a change in symptom pattern over time according to the BDS organ systems symptom clusters [9]. Our study showed that all four delimitations could develop into one of the three others. This interchange between syndromes was very frequent and should be considered in future studies. When not taking this into account, reported incidence, remission or persistence might reflect change from one type of FSD to another.

In general, the available literature is limited with several suggested delimitations, ranging from persons self-perceived diagnoses over data from administrative registries to various delimitations based on questionnaires. As such, a major issue in the research area is the lack of transparent diagnostic criteria and usage of data that was not initially meant for studying FSD. New cohorts like DanFunD emerge [35] and it is necessary to initiate further similar cohort studies with the focus on unraveling the epidemiology of FSD. As such, the investigation of predictors of FSD has finally reached a crucial point with comprehensive longitudinal studies such as the DanFunD-cohort and Lifelines [6] providing opportunities to explore both biological, psychological, and sociological determinants of FSD.

### Strengths and limitations

The major strengths of the DanFunD study include that the primary focus of the study was to unravel the epidemiology of FSD. It represents a large population-based cohort design, longitudinally assessing FSD at two time points (median 65 months apart) allowing for assessment of incidence, persistence, and remission. The thorough symptom-based assessment of FSD at two time points using transparent diagnostic criteria allows for further studies on causality of FSD, which has been lacking for researchers and clinicians. The assessment of multiple delimitations of FSD is a major strength of our study, as this enables us to investigate commonalities and differences in the delimitations and taking overlap into account.

A limitation of the study is that persons with FSD and middle-aged persons had a lower attendance at follow-up, which can introduce selection bias and limit the internal validity of the study. Similar tendencies were seen in a prospective study in primary care on BDS-prognosis [9]. These conditions are similar for all longitudinal studies relying on self-report or interview of participants.

Nonetheless the reliance on self-reported symptoms carries a risk of misclassification of FSD as comorbid diseases may share symptoms with FSD and could result in the reported symptoms. To limit this risk, only bothersome symptoms within 12 months are considered for the definitions [27]. A study comparing the agreement between BDS diagnoses based on the DanFunD symptom questionnaire and diagnostic interviews found a lower prevalence of FSD in interview-based diagnoses. This was mainly due to “the interviewer’s evaluation and distinction between FSD symptom patterns and symptoms caused by other relevant conditions” [36]. As such, the reported exact incidences, remissions, and persistence must be interpreted with caution as they are at risk of overestimation due to the categorizations based on self-reports. Yet, the large scale, comprehensive cohort design, and long follow-up time allow for findings of valuable epidemiological and etiological tendencies on the natural history and the global picture of the fluctuations in FSD. This finding is important, as we know little about the natural course of these syndromes, and therefore our study provides a solid epidemiological framework for future studies.

## Conclusion

This population-based follow-up study has shown a marked fluctuation pattern of FSD during a five-year period with high and variating incidence and remission proportions. Furthermore, the various FSS show a high degree of interchangeability between each other. The study stresses the importance of large population-based cohorts with transparent delimitation of the most common FSD in future research in understanding these complex conditions.

## Supporting information

S1 Fig Flowchart for definitions of incidence, remission, and persistence of bodily distress syndrome. (TIF)

S2 Fig Flowchart for definitions of incidence, remission, and persistence of functional somatic syndromes. (TIF)
