# Supplementary figures and images for: Fluctuation of functional somatic disorders in a population-based cohort. The DanFunD study
Source: PLoS One. 2024 Oct 16;19(10):e0312031. doi: 10.1371/journal.pone.0312031 (PMC11482674; doi:10.1371/journal.pone.0312031)

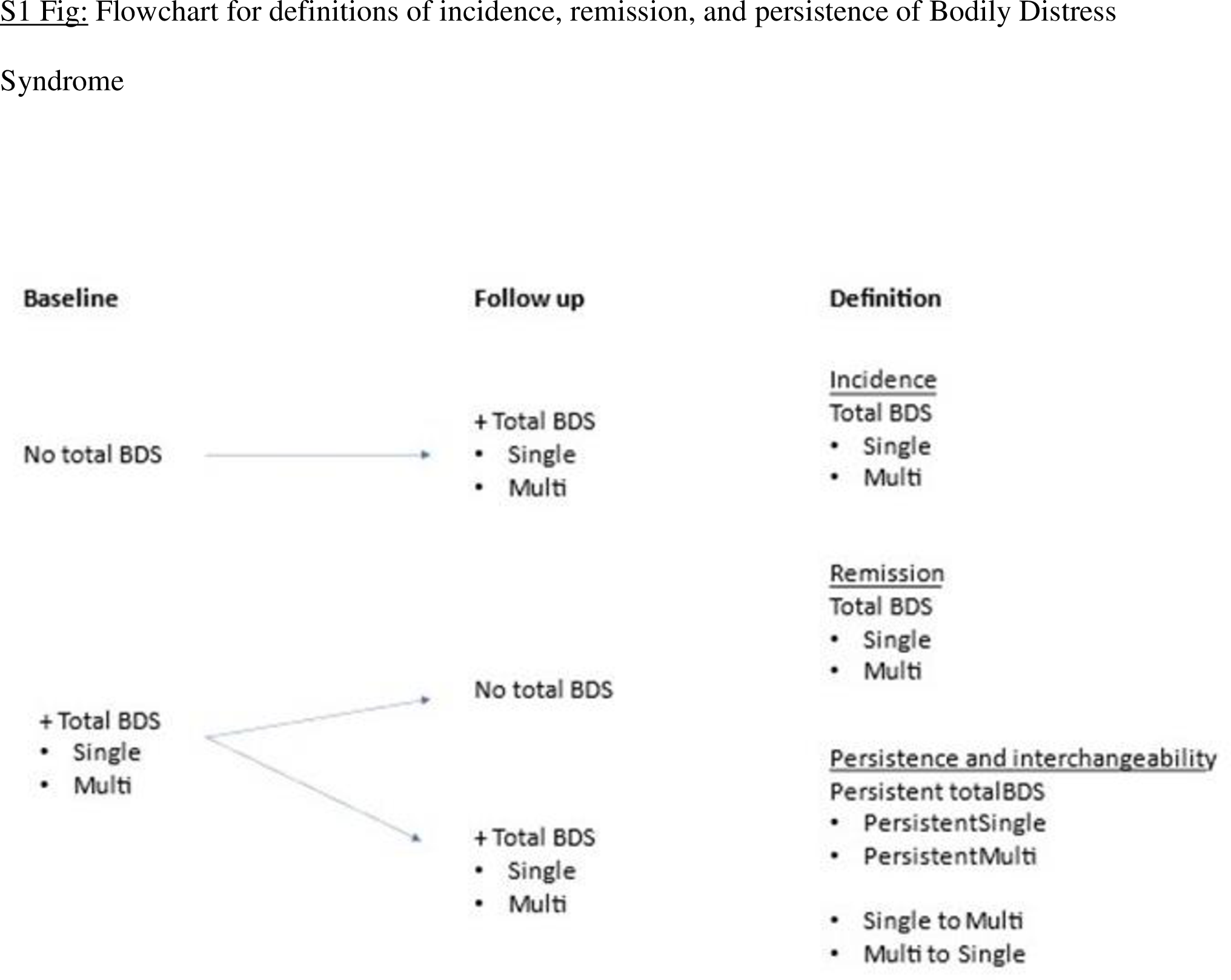

Supplement: S1 Fig — (TIF) [file pone.0312031.s001.tif]

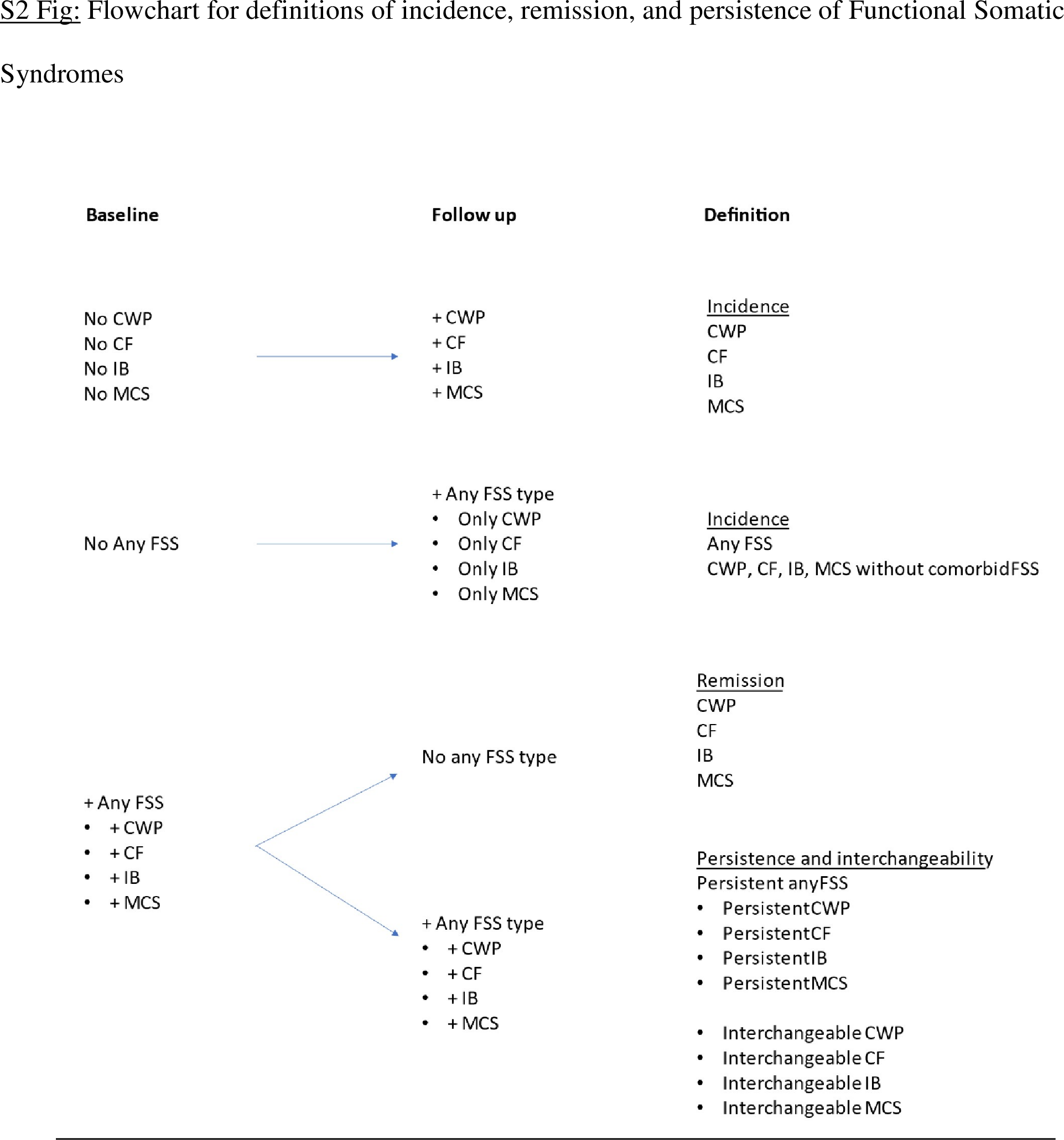

Supplement: S2 Fig — (TIF) [file pone.0312031.s002.tif]
